# Choosing the right rehabilitation setting after herniated disc surgery: Motives, motivations and expectations from the patients’ perspective

**DOI:** 10.1371/journal.pone.0183698

**Published:** 2017-08-22

**Authors:** Margrit Löbner, Janine Stein, Melanie Luppa, Alexander Konnopka, Hans Jörg Meisel, Lutz Günther, Jürgen Meixensberger, Katarina Stengler, Matthias C. Angermeyer, Hans-Helmut König, Steffi G. Riedel-Heller

**Affiliations:** 1 Institute of Social Medicine, Occupational Health and Public Health, University of Leipzig, Leipzig, Germany; 2 Department of Health Economics and Health Services Research, University Medical Center Hamburg-Eppendorf, Hamburg, Germany; 3 Department of Neurosurgery, Berufsgenossenschaftliche Kliniken Bergmannstrost, Halle (Saale), Germany; 4 Department of Neurosurgery, Klinikum St. Georg gGmbH, Leipzig, Germany; 5 Department of Neurosurgery, University of Leipzig, Leipzig, Germany; 6 Department of Psychiatry, Psychosomatic Medicine and Psychotherapy, HELIOS Park Hospital Leipzig, Leipzig, Germany; 7 Center for Public Mental Health, Gösing am Wagram, Austria; 8 Department of Public Health, University of Cagliary, Cagliary, Italy; University of Illinois at Urbana-Champaign, UNITED STATES

## Abstract

**Objectives:**

This study aims to investigate (1) motives, motivations and expectations regarding the choice for a specific rehabilitation setting after herniated disc surgery and (2) how rehabilitation-related motivations and expectations are associated with rehabilitation outcome (ability to work, health-related quality of life and satisfaction with rehabilitation) three months after disc surgery.

**Methods:**

The longitudinal cohort study refers to 452 disc surgery patients participating in a subsequent rehabilitation. Baseline interviews took part during acute hospital stay (pre-rehabilitation), follow-up interviews three months later (post-rehabilitation). Binary logistic regression and multiple linear regression analyses were applied.

**Results:**

(1) Motives, motivations and expectations: Inpatient rehabilitation (IPR) patients stated “less effort/stress” (40.9%), more “relaxation and recreation” (39.1%) and greater “intensity of care and treatment” (37.0%) regarding their setting preference, whereas outpatient rehabilitation (OPR) patients indicated “family reasons” (45.3%), the wish for “staying in familiar environment” (35.9%) as well as “job-related reasons” (11.7%) as most relevant. IPR patients showed significantly higher motivation/expectation scores regarding regeneration (p < .001), health (p < .05), coping (p < .001), retirement/job (p < .01), psychological burden (p < .05) and physical burden (p < .001) compared to OPR patients. (2) Associations with rehabilitation outcome: Besides other factors (e.g. age, gender and educational level) rehabilitation-related motivations/expectations were significantly associated with rehabilitation outcome measures. For example, patients with less motivations/expectations to achieve improvements regarding “physical burden” showed a better health-related quality of life (p < .01) three months after disc surgery. Less motivations/expectations to achieve improvements regarding “psychological burden” was linked to a better mental health status (p < .001) and a greater satisfaction with rehabilitation (OR = .806; p < .05).

**Conclusion:**

Rehabilitation-related motivations and expectations differed substantially between IPR and OPR patients before rehabilitation and were significantly associated with rehabilitation outcome. Taking motivational and expectation-related aspects into account may help to improve allocation procedures for different rehabilitation settings and may improve rehabilitation success.

## Introduction

Musculoskeletal disorders are the major indication group for a rehabilitation treatment in Germany [1], including rehabilitation treatments after herniated disc surgery. In Germany, patients undergoing herniated disc surgery are recommended to continue their treatment in a rehabilitation facility immediately (within two weeks) after acute hospital care. This subsequent rehabilitation (“Anschlussheilbehandlung”, AHB) generally takes about 3 weeks, but can be extended if medically necessary. The procedure is here as follows: the attending physician in acute care hospital determines the need for rehabilitation, thereafter the hospital social service assists the patient with filling in and forwarding the AHB application to the patients´ responsible institution for the AHB costs. The responsible institution for the AHB costs (normally the patients`health insurance or pension insurance company) is also in charge of the final decision regarding the acceptance of AHB and the AHB facility.

According to Paragraph 9 of the German Social Code IX (SGB IX) patients who apply for AHB have a “Wunsch- und Wahlrecht” meaning “the right to individual wishes and choice relative to assessments, services and institutions as well as to the various benefits…concerning every aspect of the implementation of these services.” [2]. This also implies that patients have the right to individual wishes and choice regarding the rehabilitation setting: inpatient or outpatient. However, Pohontsch et al. found that most patients in their study neither know about their right to individual wishes and choice of rehabilitation setting nor get informed about this right during application process. Nonetheless, the aim of such a “Wunsch- und Wahlrecht” is to support self-efficacy and personal responsibility of the individual during the rehabilitation process, which may have major implications for the rehabilitation-related motivation [2].

A successful rehabilitation outcome after medical conditions does not only depend on the severity of illness, nor the quality of the rehabilitation treatment itself. The achievement of a rehabilitation programme is also linked to the individuals`motivation and compliance regarding the rehabilitation process [3]. Finding the perfect fit between the patients`need and rehabilitation facility may also rely on the chosen rehabilitation setting. To date little research has been done to investigate patient-related decision processes regarding the rehabilitation setting. However, an examination of rehabilitation-related motives, motivations and expectations may hold important information for rehabilitation research regarding shared decision making and self-management [4]. Accordingly, it may also have major implications for the improvement of self-efficacy and personal responsibility of rehabilitation patients in both inpatient and outpatient settings. The present study makes a first attempt to address this matter for patients participating in subsequent rehabilitation (AHB) after herniated disc surgery by examining the following questions:

What are motives, motivations and expectations regarding the choice for a specific rehabilitation setting (inpatient or outpatient) after herniated disc surgery?

How are rehabilitation-related motivations and expectations associated with rehabilitation outcome (ability to work, health-related quality of life and satisfaction with rehabilitation) three months after disc surgery?

## Material and methods

### Study design and population

This study is a longitudinal cohort study. Detailed information on study design and sample is published elsewhere [5]. A total of 534 consecutive herniated disc surgery patients (response rate: 86%) participated in a baseline interview (T0) within acute care hospital. The patient recruitment took place at three neurosurgery departments in Central Germany: Hospital St. Georg Leipzig (N = 153), University Hospital Leipzig (N = 150) and Hospital Bergmannstrost Halle (Saale) (N = 231). Three months later 486 nucleotomy patients also participated in a telephone follow-up survey (T1) (dropout rate: 9%). This paper only focusses on patients who have participated in an AHB program between acute hospital stay (T0) and follow-up survey (T1). Thus, the presented data refer to 452 study patients. Out of those 307 patients attended an inpatient rehabilitation setting (IPR) and 145 patients an outpatient setting (OPR) [5].

### Ethics statement

The study has received ethics committee approval of the University of Leipzig (Ethik-Kommission an der Medizinischen Fakultät der Universität Leipzig). At the initial contact, participants were verbally informed on the purpose of the study (including handing out a study information form) and provided written consent to take part in the study.

### Baseline properties (T0)—Before rehabilitation

#### Socio-demographic characteristics

This study contains information about age, gender, family status and educational level.

#### Illness-related and psychological characteristics

Besides the length of acute care hospital stay (when disc surgery was conducted), disc location (lumbar versus cervical) and the presence of other chronic diseases were assessed. Pain intensity was measured by using a verbal numeric pain scale (range: 0–100, higher scores indicate more severe pain). The Composite International Diagnostic Interview (CIDI-DIA-X, computerized version) [6] was used to assess 4-week prevalences of psychiatric comorbidity (affective, anxiety and substance-related disorders).

#### Vocational characteristics

Patients were asked whether they were employed within the last 3 months before baseline and for their subjective prognosis of gainful employment (SPE-scale) [7,8]. The SPE-scale contains 3 items (range: 0–3, higher scores indicate a worse subjective prognosis of gainful employment).

#### Rehabilitation-related characteristics

The baseline interview included the FREM-17 [9] (questionnaire for assessing rehabilitational expectations and motivations) and the PAREMO-20 [10] (patient questionnaire for assessment of rehabilitation motivation). Both instruments determine the general rehabilitation-related motivation of a patient and have been proved to be practicable, valid and reliable instruments [3,11]. The FREM-17 and the PAREMO-20 are both multidimensional instruments that can be used for different clinical indications [3,11]. As a main difference both questionnaires form different thematic dimensions of rehabilitation-related motivations. In addition, while the FREM-17 questions the individual expectations towards rehabilitation treatment as an indicator for motivation [3, 11], the PAREMO-20 rather gives information about favourable and unfavourable pre-rehabilitational conditions in order to draw conclusions about rehabilitation motivation of an individual [10]. The FREM-17 consists of 17 items forming the following four domains: “regeneration” (five items), “health” (four items), “coping” (four items) and “retirement/job” (four items). The PAREMO-20 consists of 20 items forming six subscales: “psychological burden” (three items), “physical burden” (four items), “social support” (four items), “readiness to change” (three items), “knowledge” (3 items) and “scepticism” (three items). Higher values on the first five subscales are considered to indicate higher rehabilitation motivation. The subscale “scepticism” is an exception: higher scores rather indicate lower rehabilitation motivation. In order to obtain the highest possible amount of information the present study refers to the results of both instruments. Also at baseline, patient’s motives to prefer either inpatient or outpatient rehabilitation were assessed with a qualitative approach (qualitative content analysis, Mayring 2000 [12]). Using an “open-ended” question patients were hereby asked to explain their individual motives behind their preference for a certain rehabilitation setting (inpatient vs. outpatient). Thus, multiple responses were possible. Motive categories for specific rehabilitation setting preferences were composed following the step model of inductive category development (Mayring, 2000 [13]). As a last step, frequencies of the coded motive categories were analysed. Analyses only comprise data of patients whose setting preferences at baseline and the actual setting attendance until follow-up interview was compatible.

### Follow-up properties (T1)—After rehabilitation

#### Rehabilitation-related characteristics

Within follow-up survey it was investigated whether patients took part in an “inpatient”, “outpatient” or “no” subsequent rehabilitation between baseline and follow-up survey.

#### Ability to work

Additionally, patients were asked whether they recovered their ability to work (ATW), or were still on certified sick leave or were already receiving an early retirement pension (NATW).

#### Health-related quality of life

The Short Form 36 Health Survey (SF-36) was used to assess health-related quality of life [14]. The reliable, valid and responsive instrument [15–18] comprises 36 items which sum up to eight subscales of functional health and well-being scores. The eight subscales can be further consolidated into a mental component summary score (MCS) further referred to as mental health status (including the subscales vitality, social functioning, role limitations due to emotional problems and mental health) and a physical component summary score (PCS) further referred to as physical health status (including the subscales physical functioning, role limitations due to physical health problems, bodily pain, general health) [14]. The scores of each component summary score range from 0 to 100, with higher scores indicating better health [14].

#### Satisfaction with rehabilitation

Patients were asked about their overall satisfaction with rehabilitation using a 5 point Likert-Scale which was later on dichotomized into a binary satisfaction variable: “yes” (100% satisfied, very satisfied, satisfied) and “no” (rather unsatisfied, unsatisfied).

### Statistical methods

All calculations were carried out using the Statistical Package for the Social Sciences (SPSS) Version 20.0 [19] and STATA Version 13 [20]. The significance level was set at α = 0.05 for all statistical analyses. Independent T-tests as well as Chi-Square-tests were used to analyse differences regarding rehabilitation-related motives, motivations and expectations in both patient groups (IPR vs. OPR). The chosen rehabilitation outcome parameters “ability to work” and “satisfaction with rehabilitation”were analysed using binary logistic regression models (enter method). Two multiple linear regression analyses were applied in order to investigate the rehabilitation outcome parameter “health-related quality of life” (physical and mental health status). Socio-demographic, illness-related, vocational and rehabilitation-related characteristics were included as independent variables within all regression models. Besides the attended rehabilitation setting (IPR vs. OPR) also rehabilitation-related motivations and expectations were taken into account.

## Results

### Age and gender distribution in IPR and OPR patients

A detailed comparison of socio-demographic, illness-related and work-related characteristics in IPR and OPR patients is published elsewhere [5]. Accordingly, OPR patients were significantly younger than IPR patients (mean (SD) OPR = 40.7 (8.4); mean (SD) IPR = 43.4 (7.3); p < .01) [5]. No significant differences could be found regarding the gender distribution in both groups (OPR: 37.2% females/62.8% males versus IPR: 45.3% females/ 54.7% males) [5].

### Motives, motivations and expectations regarding the choice for a specific rehabilitation setting

Table 1 shows the results regarding rehabilitation-related motivations and expectations within both rehabilitation settings. IPR patients showed significantly higher motivation and expectation scores regarding regeneration (T = 10.474; df = 240.745; p < .001), health (T = 2.397; df = 450; p < .05), coping (T = 4.148; df = 449; p < .001), retirement/job (T = 2.747; df = 450; p < .01), psychological burden (T = 2.686; df = 386.048; p < .05) and physical burden (T = 3.561; df = 450; p < .001). No significant differences regarding motivation and expectation scores could be found for social support/reactions of significant others to the illness, readiness to change, scepticism and knowledge.

**Table 1:** Comparison of rehabilitation-related motivations and expectations in patients preferring inpatient or outpatient rehabilitation setting.

Instrument	Scale	N_IPR_/N_OPR_	IPR patients		OPR patients			
			mean (SD)	[min;max]	mean (SD)	[min;max]	p-value	
**FREM-17** ^a^	regeneration	307/145	13.8 (3.9)	[0;18]	9.0 (4.8)	[0;18]	**.000**	***
	health	307/145	10.0 (2.1)	[2;12]	9.5 (2.3)	[0;12]	**.017**	*
	coping	306/145	7.4 (3.5)	[0;12]	5.9 (3.8)	[0;12]	**.000**	***
	retirement/job	307/145	3.4 (3.3)	[0;9]	2.5 (3.2)	[0;9]	**.006**	**
**PAREMO-20** ^b^	psychological burden	307/144	4.5 (2.3)	[3;12]	4.0 (1.6)	[3;10]	**.018**	*
	physical burden	307/145	12.9 (2.7)	[4;16]	11.9 (3.0)	[4;16]	**.000**	***
	social support/ reactions of significant others to the illness	306/145	11.2 (3.3)	[4;16]	11.3 (3.3)	[4;16]	.784	
	readiness to change	306/144	7.0 (2.9)	[3;12]	6.7 (2.6)	[3;12]	.191	
	skepticism	306/144	4.5 (2.0)	[3;12]	4.3 (1.9)	[3;12]	.393	
	knowledge	307/145	9.1 (3.1)	[3;12]	8.7 (3.0)	[3;12]	.296	

Calculations via independent T-tests (* p < .05, ** p < .01, *** p < .001);

IPR, inpatient rehabilitation patients;

OPR, outpatient rehabilitation patients;

N_IPR_/N_OPR_, number of inpatient rehabilitation patients/number of outpatient rehabilitation patients;

^a^ FREM-17, questionnaire for assessing rehabilitational expectations and motivations;

^b^ PAREMO-20, patient questionnaire for assessment of rehabilitation motivation

**Fig 1:**
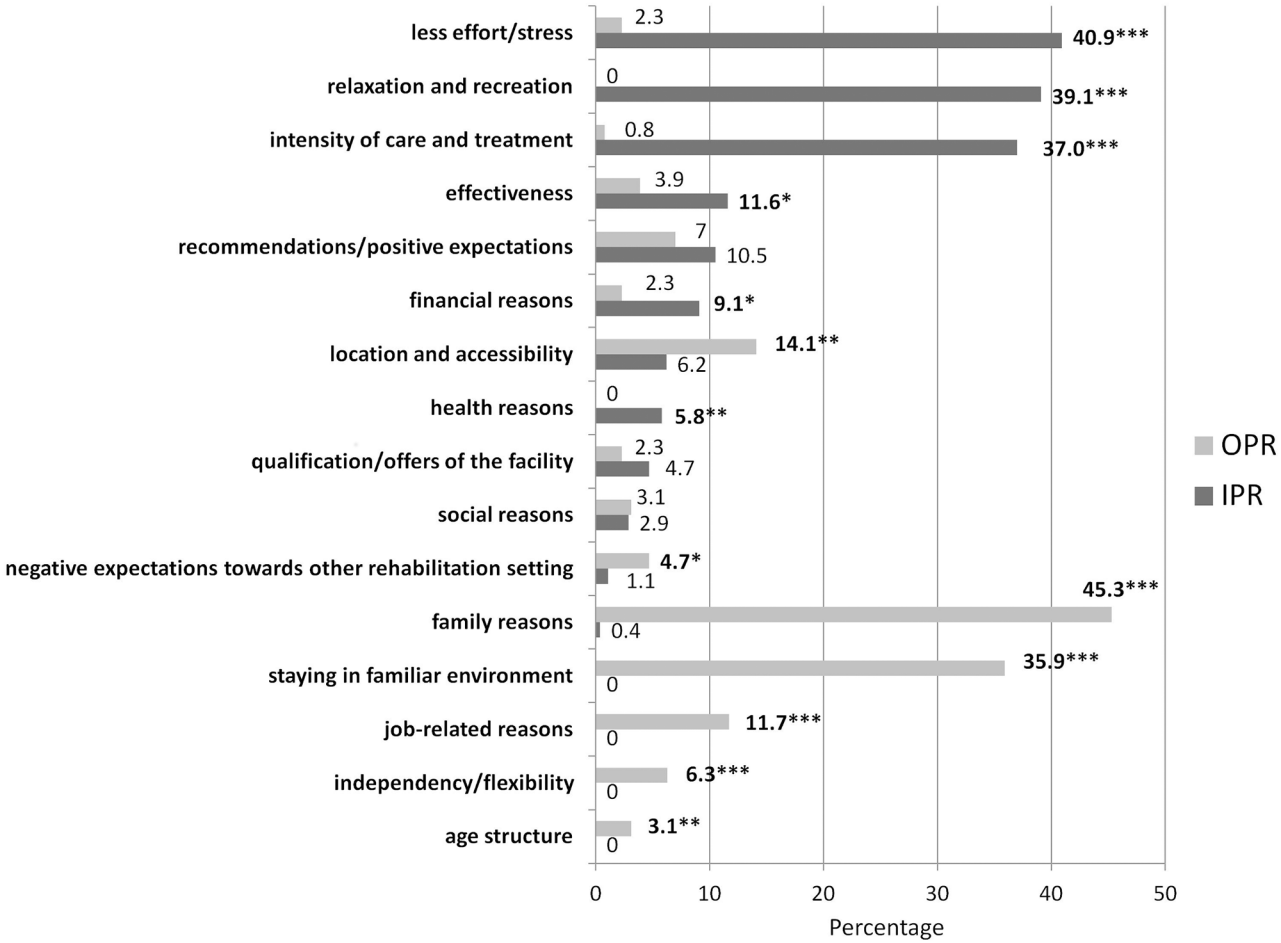
Patients´ motives for preferring a certain rehabilitation setting at baseline (T0). Calculations via Chi-Square-Tests; * p < .05, ** p < .01, *** p < .001; IPR, inpatient rehabilitation patients; OPR, outpatient rehabilitation patients; analyses only comprise data of patients whose setting preferences at baseline and the actual setting attendance until Follow-up interview are compatible.

The qualitative approach to find individual motives behind patients´ preferences for a certain rehabilitation setting (inpatient vs. outpatient) revealed 16 motive categories which are presented in Fig 1. 276 (89.9%) of the 307 patients who actually attended rehabilitation treatment in an IPR facility provided qualitative data about motives behind their setting preferences at baseline interview. Out of those 130 patients (47.1%) stated only one reason and 146 patients (52.9%) two or more reasons for preferring IPR. The three most frequently mentioned motives for preferring inpatient rehabilitation setting were, that the rehabilitation stay was connected with “less effort/stress” (40.9%), more “relaxation and recreation” (39.1%), and a greater “intensity of care and treatment” (37.0%). 128 (88.3%) of the 145 patients who actually attended rehabilitation treatment in an OPR facility provided qualitative data about motives behind their setting preferences at baseline interview. Out of those 85 patients (66.4%) stated only one reason and 43 patients (33.6%) two or more reasons for preferring OPR. The three most frequently named motives for preferring outpatient rehabilitation setting were, “family reasons” (45.3%), the wish for “staying in familiar environment” (35.9%), as well as “job-related reasons” (11.7%).

### Ability to work at follow-up (T1)

All patients (N = 452) who participated in subsequent rehabilitation provided information about their ability to work status at follow-up interview. Thus, ability to work rates were significantly higher in OPR patients (62.8%) compared to IPR patients (29.6%) (Chi2 = 44.907; df = 1; p < .001). The results of binary logistic regression analysis (Table 2) show that higher age (OR = 1.041; p < .05), a better educational level (OR = 3.753; p < .05), the absence of other chronic diseases (OR = .586; p < .05), a shorter length of acute care hospital stay (OR = .848; p < .01), a better subjective prognosis of gainful employment (OR = .692; p < .05) and outpatient rehabilitation treatment (OR = 4.277; p < .001) were associated with higher ability to work rates three months after disc surgery. Rehabilitation-related motivations/expectations did not prove to have a significant association with ability to work.

**Table 2:** Binary logistic regression model of factors associated with ability to work^a^ three months after disc surgery (N = 428^b^).

Predictors of ability to work		B	*p*	OR	95% CI (OR)		
**Sociodemographic characteristics**							
gender	male (R:female)	.294	.227	1.342	.832	-	2.164
age		.040	.026*	1.041	1.005	-	1.079
family status	R:single						
	married	-.008	.980	.992	.538	-	1.831
	seperated/divorced/widowed	-.260	.527	.771	.344	-	1.728
educational level	R: til 9th grade						
	10th grade	.745	.117	2.106	.829	-	5.352
	Abitur/ Technical college qualification/ University qualification	1.322	.013*	3.753	1.317	-	10.692
**Illness-related characteristics**							
disc location	cervical (R: lumbar)	-.431	.170	.650	.351	-	1.203
other chronic diseases	yes (R: no)	-.535	.036*	.586	.356	-	.965
psychiatric comorbidity within the last 4 weeks (CIDI-DIAX) ^1^	yes (R: no)	-.867	.070	.420	.165	-	1.073
pain intensity (pain scale, 0–100)		.002	.707	1.002	.991	-	1.014
length of hospital stay (days)		-.165	.005**	.848	.755	-	.953
**Vocational characteristics**							
employment within the last three months	yes (R: no)	-.096	.787	.908	.452	-	1.827
subjective prognosis of gainful employment (SPE-scale) ^2^		-.367	.012*	.692	.519	-	.923
**Rehabilitation-related characteristics**							
rehabilitation setting	R: inpatient						
	outpatient	1.453	.000***	4.277	2.404	-	7.607
rehabilitation-related motivations and expectations (FREM-17) ^3^	regeneration	.013	.688	1.013	.952	-	1.077
	health	.025	.688	1.025	.909	-	1.156
	coping	-.020	.612	.980	.906	-	1.060
	retirement, job	-.055	.257	.946	.860	-	1.041
rehabilitation-related motivations (PAREMO-20) ^4^	psychological burden	.094	.169	1.098	.961	-	1.255
	physical burden	-.082	.073	.921	.842	-	1.008
	social support/ reactions of significant others to the illness	-.015	.692	.985	.913	-	1.062
	readiness to change	-.002	.973	.998	.907	-	1.099
	skepticism	-.039	.594	.962	.833	-	1.110
	knowledge	-.023	.541	.977	.908	-	1.052

Nagelkerkes R^2^–0.333;

B, Regression Coefficient B;

OR, Odds Ratio;

95% CI (OR), 95% Confidence Interval of Odds Ratio;

p, p-value;

* p < .05,

** p < .01,

*** p < .001;

^a^ Reference category is non-ability to work three months after disc surgery;

^b^ analysis only included cases with non-missing values for dependent and independent variables;

^1^ CIDI, Composite International Diagnostic Interview;

^2^ SPE, Subjective prognosis of gainful employment;

^3^FREM-17, questionnaire for assessing rehabilitational expectations and motivations;

^4^ PAREMO-20, patient questionnaire for assessment of rehabilitation motivation

### Health-related quality of life at follow-up (T1)

451 (99.8%) of the patients who participated in subsequent rehabilitation provided information about their health-related quality of life at follow-up interview. OPR patients did not only report a significantly better physical health status compared to IPR (mean (SD) _OPR_ = 41.1 (11.3); mean (SD) _IPR_ = 36.3 (9.6); p < .001), but also a significantly better mental health status (mean (SD) _OPR_ = 55.8 (9.7); mean (SD) _IPR_ = 52.5 (12.5); p < .01) three months after disc surgery. The results of multiple linear regression analysis regarding the physical health status are shown in Table 3. Thus, male gender (p < .05), the absence of other chronic diseases (p < .05), lower pain intensity (p < .001), a shorter length of acute care hospital stay (p < .05), employment within the last three months (p < .01), a better subjective prognosis of gainful employment (p < .01) and outpatient rehabilitation treatment (p < .05) is significantly associated with a better physical health status. In addition, patients with less rehabilitation-related motivations/expectations regarding “physical burden” (p < .01), but more rehabilitation-related motivations/expectations regarding “psychological burden” (p < .01) are more likely to have a better physical health status. The results of multiple linear regression analysis regarding the mental health status are shown in Table 4. Accordingly, a better educational level (10th grade vs. 9th grade, (p < .01)), having surgery due to a lumbar disc herniation (vs. cervical, p < .05) and the absence of psychiatric comorbidity (p < .05) is significantly associated with a better mental health status. Additionally, patients with less rehabilitation-related motivations/expectations regarding “coping” (p < .01), “psychological burden” (p < .001), “physical burden” (p < .01) and less “scepticism” (p < .05) regarding rehabilitation treatment but more rehabilitation-related motivations/expectations regarding “health” (p < .05) are more likely to have a better mental health status.

**Table 3:** Multiple linear regression model of factors associated with physical health status (global score 0–100, SF-36) three months after disc surgery (N = 427^a^).

Predictors of physical health status		Coef.	*p*	95% CI		
**Sociodemographic characteristics**						
gender	male (R:female)	2.115	.021*	.326	-	3.904
age		-.065	.333	-.198	-	.067
family status	R:single					
	married	-.379	.751	-2.730	-	1.970
	seperated/divorced/widowed	-.151	.920	-3.111	-	2.809
educational level	R: til 9th grade					
	10th grade	-.474	.758	-3.497	-	2.549
	Abitur/ Technical college qualification/ University qualification	-.114	.949	-3.418	-	3.646
**Illness-related characteristics**						
disc location	cervical (R: lumbar)	1.764	.123	-.482	-	4.011
other chronic diseases	yes (R: no)	-2.349	.013*	-4.198	-	-.499
psychiatric comorbidity within the last 4 weeks (CIDI-DIAX) ^1^	yes (R: no)	-.982	.535	-4.092	-	2.127
pain intensity (pain scale, 0–100)		-.079	.000***	-.121	-	-.038
length of hospital stay (days)		-.401	.021*	-.743	-	-.060
**Vocational characteristics**						
employment within the last three months	yes (R: no)	3.882	.002**	1.389	-	6.374
subjective prognosis of gainful employment (SPE-scale) ^2^		-1.810	.001**	-2.874	-	-.746
**Rehabilitation-related characteristics**						
rehabilitation setting	R: inpatient					
	outpatient	2.635	.016*	.494	-	4.777
rehabilitation-related motivations and expectations (FREM-17) ^3^	regeneration	.031	.792	-.198	-	.260
	health	-.221	.326	-.664	-	.221
	coping	-.010	.946	-.308	-	.287
	retirement, job	.119	.486	-.217	-	.456
rehabilitation-related motivations (PAREMO-20) ^4^	psychological burden	.742	.002**	.267	-	1.216
	physical burden	-.583	.001**	-.925	-	-.241
	social support/ reactions of significant others to the illness	-.058	.679	-.333	-	.217
	readiness to change	.233	.191	-.117	-	.583
	skepticism	-.200	.416	-.681	-	.282
	knowledge	-.105	.463	-.385	-	.175

R^2^–0.312;

Coef., Regression Coefficient;

95% CI, 95% Confidence Interval of Regression Coefficient;

p, p-value;

* p < .05,

** p < .01,

*** p < .001;

^a^ analysis only included cases with non-missing values for dependent and independent variables;

^1^ CIDI, Composite International Diagnostic Interview;

^2^ SPE, Subjective prognosis of gainful employment;

^3^ FREM-17, questionnaire for assessing rehabilitational expectations and motivations;

^4^ PAREMO-20, patient questionnaire for assessment of rehabilitation motivation

**Table 4:** Multiple linear regression model of factors associated with mental health status (global score 0–100, SF-36) three months after disc surgery (N = 427^a^).

Predictors of mental health status		Coef.	p	95% CI		
**Sociodemographic characteristics**						
gender	male (R:female)	1.299	.189	-.640	-	3.237
age		-.095	.195	-.239	-	.049
family status	R:single					
	married	-.491	.705	-3.037	-	2.055
	seperated/divorced/widowed	-1.002	.539	-4.208	-	2.205
educational level	R: til 9th grade					
	10th grade	4.344	.009**	1.069	-	7.619
	Abitur/ Technical college qualification/ University qualification	3.295	.091	-.531	-	7.122
**Illness-related characteristics**						
disc location	cervical (R: lumbar)	-2.875	.021*	-5.309	-	-.441
other chronic diseases	yes (R: no)	1.730	.090	-.273	-	3.734
psychiatric comorbidity within the last 4 weeks (CIDI-DIAX) ^1^	yes (R: no)	-3.573	.038*	-6.942	-	-.204
pain intensity (pain scale, 0–100)		.032	.160	-.013	-	.077
length of hospital stay (days)		.273	.147	-.097	-	.643
**Vocational characteristics**						
employment within the last three months	yes (R: no)	-2.096	.128	-4.796	-	.604
subjective prognosis of gainful employment (SPE-scale) ^2^		-.182	.756	-1.335	-	.971
**Rehabilitation-related characteristics**						
rehabilitation setting	R: inpatient					
	outpatient	.952	.420	-1.368	-	3.272
rehabilitation-related motivations and expectations (FREM-17) ^3^	regeneration	-.044	.726	-.292	-	.204
	health	.586	.017*	.107	-	1.066
	coping	-.500	.002**	-.822	-	-.178
	retirement, job	.161	.385	-.203	-	.526
rehabilitation-related motivations (PAREMO-20) ^4^	psychological burden	-2.311	.000***	-2.825	-	-1.797
	physical burden	-.430	.023*	-.800	-	-.060
	social support/ reactions of significant others to the illness	-.197	.195	-.495	-	.101
	readiness to change	-.258	.183	-.637	-	.122
	skepticism	-.631	.018*	-.1.153	-	-.110
	knowledge	-.019	.901	-.323	-	.284

R^2^–0.390;

Coef., Regression Coefficient;

95% CI, 95% Confidence Interval of Regression Coefficient;

p, p-value;

* p < .05,

** p < .01,

*** p < .001;

^a^ analysis only included cases with non-missing values for dependent and independent variables;

^1^ CIDI, Composite International Diagnostic Interview;

^2^ SPE, Subjective prognosis of gainful employment;

^3^ FREM-17, questionnaire for assessing rehabilitational expectations and motivations;

^4^ PAREMO-20, patient questionnaire for assessment of rehabilitation motivation

### Satisfaction with rehabilitation at follow-up (T1)

448 of the patients (99.1%) provided information about their satisfaction with subsequent rehabilitation. Thus, the majority of the study sample recorded to be satisfied with AHB (92.8% IPR patients, 93.7% OPR patients). There were no statistically significant differences between different rehabilitation settings (Chi2 = .128; df = 1; p = .721). The results of binary logistic regression analysis (Table 5) revealed that male patients were more than twice as likely to be satisfied with AHB (OR = 2.568; p < .05) compared to women. In addition, patients with less rehabilitation-related motivations regarding “psychological burden” (OR = .823; p < .05) but more “knowledge” regarding rehabilitation treatment (OR = 1.182; p < .05) were more likely to be satisfied with AHB.

**Table 5:** Binary logistic regression model of factors associated with satisfaction with rehabilitation^a^ three months after disc surgery (N = 424^b^).

Predictors of satisfaction with rehabilitation		B	*p*	OR	95% CI (OR)		
**Sociodemographic characteristics**							
gender	male (R:female)	.943	.040*	2.568	1.043	-	6.325
age		.029	.421	1.030	.959	-	1.106
family status	R:single						
	married	-.097	.878	.908	.262	-	3.140
	seperated/divorced/widowed	-.958	.156	.384	.102	-	1.440
educational level	R: til 9th grade						
	10th grade	.096	.904	1.100	.235	-	5.159
	Abitur/ Technical college qualification/ University qualification	-.197	.821	.882	.150	-	4.491
**Illness-related characteristics**							
disc location	cervical (R: lumbar)	-.698	.182	.497	.178	-	1.388
other chronic diseases	yes (R: no)	.689	.159	1.991	.764	-	5.190
psychiatric comorbidity within the last 4 weeks (CIDI-DIAX) ^1^	yes (R: no)	-1.154	.062	.315	.094	-	1.059
Pain intensity (pain scale, 0–100)		-.011	.246	.989	.970	-	1.008
length of hospital stay (days)		-.083	.295	.920	.787	-	1.075
**Vocational characteristics**							
employment within the last three months	yes (R: no)	-.354	.590	.702	.193	-	2.548
subjective prognosis of gainful employment (SPE-scale) ^2^		-.287	.283	.751	.445	-	1.267
**Rehabilitation-related characteristics**							
rehabilitation setting	R: inpatient						
	outpatient	.090	.876	1.094	.353	-	3.395
rehabilitation-related motivations and expectations (FREM-17) ^3^	regeneration	.037	.524	1.038	.925	-	1.165
	health	-.059	.624	.943	.744	-	1.194
	coping	.074	.354	1.076	.921	-	1.258
	retirement, job	.149	.112	1.161	.966	-	1.396
rehabilitation-related motivations (PAREMO-20) ^4^	psychological burden	-.216	.042*	.806	.654	-	.993
	physical burden	-.008	.934	.992	.828	-	1.190
	social support/ reactions of significant others to the illness	.012	.868	1.012	.879	-	1.165
	readiness to change	.062	.487	1.064	.893	-	1.269
	skepticism	-.087	.432	.917	.739	-	1.138
	knowledge	.167	.017*	1.182	1.030	-	1.355

Nagelkerkes R^2^–0.250;

B, Regression Coefficient B;

OR, Odds Ratio;

95% CI (OR), 95% Confidence Interval of Odds Ratio;

p, p-value;

* p < .05;

^a^ Reference category is dissatisfaction with rehabilitation three months after disc surgery;

^b^ analysis only included cases with non-missing values for dependent and independent variables;

^1^ CIDI, Composite International Diagnostic Interview;

^2^ SPE, Subjective prognosis of gainful employment;

^3^ FREM-17, questionnaire for assessing rehabilitational expectations and motivations;

^4^ PAREMO-20, patient questionnaire for assessment of rehabilitation motivation

## Discussion

The role of patients´ motivations and expectations is an under-researched topic [21], even though it has been stressed to be an important prognostic factor for the success of medical rehabilitation [9,21–23]. In addition, motivational aspects regarding rehabilitation are of great importance with regard to individual treatment planning [24]. The focus of this paper was to investigate patients`motives, motivations and expectations regarding the choice of a specific rehabilitation setting (inpatient versus outpatient) and to examine how rehabilitation-related motivations and expectations are associated with rehabilitation outcome three months after disc surgery.

Patients´ motivations and expectations before rehabilitation differed greatly depending on the specific rehabilitation setting. Thus, IPR patients had more expectations and therefore were motivated to achieve greater improvements regarding regeneration, health, coping, retirement/job, psychological burden and physical burden compared to OPR patients. A possible explanation for this difference in motivations and expectations might be a “pre-selection” of patients with worse health status into IPR [5]. Hence, the level of suffering might be much higher in patients with a worse health status, which might result in higher motivations and expectations with regard to medical rehabilitation. In line with these findings, the assessment of individual motives of patients to prefer either outpatient or inpatient rehabilitation also revealed different focal points in both groups. Whereas IPR patients indicated “less effort/stress”, more “relaxation and recreation” and a greater “intensity of care and treatment” as the three main motives for their choice, OPR patients referred to “family reasons”, the wish for “staying in familiar environment”, as well as “job-related reasons” as their main reasons. These findings imply that not only patients`health status determines the choice for a certain rehabilitation setting. Additionally, patients´ living situation, such as family-related responsibilities like child care or assisting elderly relatives seem to have an impact on setting-specific preferences. Furthermore, from patients´ perspective, an outpatient rehabilitation setting seems to provide better conditions in order to maintain job-related activities. In addition, IPR patients reported significantly more often that they believed the treatment within their rehabilitation setting to be more effective. Also, IPR patients justified their choice significantly more often with “recommendations/positive expectations”, “financial reasons” (e.g. less travel costs) and “health reasons” (e.g. not being able to manage daily drives to OPR setting for health reasons). It is an interesting fact, that IPR patients were more likely to state a better “effectiveness” as a reason to prefer inpatient setting. It is conceivable that more detailed information about similarities of rehabilitation treatments and effectiveness in each of the two settings may influence this motive of choice. Future studies are needed to investigate the level of information before rehabilitation treatment in order to find out whether a lack of information could play a role in decision making processes.

In contrast to this, OPR patients stated significantly more often a better “location and accessibility” of the rehabilitation clinic, “negative expectations towards the other setting”, “independency/flexibility” and “age structure” (similar age) as important reasons for their choice. Of course the availability of outpatient rehabilitation facilities within urban areas is much higher compared to rural areas. Hence, it makes sense that the choice of a certain rehabilitation setting is also dependent on the local conditions. OPR patients were significantly younger than IPR patients in the present study. Hence, younger patients might prefer to stay independent and flexible rather than to focus on relaxation and recreation. They also might prefer a younger patient population within OPR setting for this reason.

The present findings suggest that gaining knowledge about patients´ motives regarding their setting preferences may help to find the most perfect fit between individuals`expectations and the chosen rehabilitation setting.

Results regarding rehabilitation outcome three months after disc surgery were drawn from examinations of ability to work status, health-related quality of life and patients´ satisfaction with rehabilitation. While there were no setting-specific group differences regarding rehabilitation satisfaction, IPR and OPR patients differed significantly regarding ability to work rates and physical as well as mental health status. Thus, OPR patients´ ability to work rate was twice as high as in IPR patients. Consistently, OPR patients reported significantly better physical and mental quality of life scores than IPR patients. In addition to this, OPR turned out to be a positive predictor variable for ability to work and a better physical health status three months after disc surgery. These findings should not be misinterpreted as quality differences between both settings. In fact these differences can be explained by greater physical as well as mental impairments prior to rehabilitation treatment [5]. In line with this, ability to work was weakly associated with the absence of other chronic diseases and a shorter length of acute hospital stay. This is also in agreement with Lillefjell et al. [25] who found that a better overall health is an important priority area to improve ability to work. Their study examined 143 patients with musculoskeletal pain who participated in a 5 week multidisciplinary rehabilitation program [25]. The overall aim of the study was to investigate outcome predictors of work ability. While the extent to which pain was experienced as troublesome (“pain experience”) was found to be an important predictor of ability to work in the study of Lillefjell [25], the worst imaginable physical pain (“pain severity”) was not found to be useful predictor variable in their sample. In line with the latter finding, “pain intensity” did not turn out to be a significant predictor for ability to work in the present study. In addition, within the present study psychiatric comorbidity was not found to be a significant predictor of ability to work three months after disc surgery, which is in line with Lillefjell et al. [25] showing no association between depression and ability to work. In contradiction to this, Lillefjell et al. [25] found anxiety to be one of the strongest predictors. A possible explanation for this might be a rather methodological difference between the two studies, as Lillefjell et al. [25] used a dimensional measure to assess depression and anxiety, whereas the present study gives results of a categorical measure of psychiatric comorbidity. Thus, anxiety symptoms might be an important negative influencing factor for regaining ability to work after rehabilitation, even though diagnosis criteria of an anxiety disorder do not have to be fulfilled.

Lindell et al. [26] found low prior sick-listing (including all diagnoses), high self-prediction (the patients´ own belief to return to work) and young age as high predictor variables for stable return to work within their sample of 125 non-acute non-specific spinal pain patients. Their results regarding the patient´s own belief to return to work goes well in line with the results of other studies [27,28]. Accordingly, also the present study is showing that a better subjective prognosis of gainful employment is weakly associated with a higher ability to work rate after disc surgery. In the same context Iles et al. [29] conducted a systematic literature review regarding the predictive value of recovery expectations for activity limitation outcomes (such as return to work) in patients with non-chronic non-specific low back pain. The results of their review imply that recovery expectation is a consistent predictor of activity limitation [29]. The findings also show that recovery expectations have the strongest prediction in case the expectation measure is time-based and specific regarding the predictable outcome [29]. These findings suggest how important it could be to assess patients´ self-prediction regarding their ability to work within rehabilitation care on a regular basis. Thus, the results of such an assessment could help to identify a high risk group for non-return to work and could have major implications for the focus of rehabilitation treatment itself.

Unlike to the results of Lillefjell et al. [25] and Lindell et al. [26] the findings of the present study suggest a weak association of higher age with a better ability to work. We are not quite sure how to explain this discrepancy. Lillefjell et al. [25] are dealing with patients suffering from chronic back pain without manifested organic disease, Lindell et al. [26] with patients suffering from non-acute and non-specific spinal pain. Patients of younger age might have a shorter medical history of pain. Thus, they might benefit much more from rehabilitation and furthermore return to work much faster than older patients with a longer pain history. The patient sample in the present study had just undergone surgery due to a herniated disc. There might be differences in severity of illness or surgery outcome between younger and older age groups that might explain this contradiction in results. Another, more methodological explanation could be that the present study refers to a rather young to medium age study population (age range: 18 to 55 years, mean age _OPR_ = 40.7 years, mean age _IPR_ = 43.4 years). This may be leading to differing results with regard to other studies.

Furthermore, a better educational level was showing a weak association with ability to work three months after disc surgery. Dionne et al. [30] hypothesize in their review regarding formal education and back pain that patients with lower education possibly have less access to some specialized interventions, might be waiting longer for consulting or have lower compliance with health professionals´ recommendations. This might be a possible explanation for the positive association of higher educational level with ability to work in the present study. Their review findings suggest a strong association of low education with longer duration and/or higher recurrence of back pain, and also that the course of a back pain episode is less favourable among persons with lower education [30]. Yet, the authors point out that there is currently only limited available evidence regarding the relationship of lower education and the outcome of interventions among back pain patients, which should be taken into account by future studies [30].

Regarding physical quality of life three months after disc surgery the findings of the presented study show that lower pain intensity and a shorter length of acute care hospital stay are weakly associated with a better physical health status. Moreover, the absence of other chronic diseases at the time of acute hospital stay was moderately associated with a better physical health status. A better overall health status at the time of disc surgery therefore seems to be important in order to predict a better physical health status three months after disc surgery. In line with Johansson et al. [31], women tend to have a higher risk for lower quality of life after disc surgery in the presented study. The authors assume that this may be due to the fact that women generally report lower scores of quality of life compared to men [31]. In line with this, Cherepanov et al. reported similar gender differences in health-related quality of life between adult women and men based on four US nationally representative data sets [32]. They found that these differences may partly be explained by sociodemographic differences and differences in socioeconomic status (age, race, marital status, education and income). Adjusting for these differences was found to reduce the gender differences regarding health-related quality of life. In addition, Emry et al. [33] found that women with cardiac disease indicated significantly lower quality of life than men with cardiac disease. Their results suggest that women with cardiac disease had a more negative subjective experience with their disease compared to men. Furthermore, Emry et al. [33] found evidence that perceived social support, especially the feeling of companionship was influencing quality of life among women. The authors suggest that providing support groups or other mechanisms that facilitate a sense of camaraderie and belonging could increase feelings of companionship and thus increase quality of life among women in this indication group. The transferability of these results to other indication and patient groups could be of great interest and should therefore be investigated within future studies. With regard to a better physical health status a strong association was additionally found for employment within the last three months and a moderate association for a better subjective prognosis of gainful employment. Thus, employment and a positive expectation regarding return to work seem to be important predictors for health-related quality of life three months after disc surgery. Johansson et al. [31] who found similar results even argue that it might be of great importance to identify a risk group for unfavourable surgery outcome by assessing patient´s beliefs about their future work capacity. Again, rehabilitation-related motivations and expectations showed weak associations in this context. Hence, patients with fewer expectations and therefore less motivation to achieve improvements regarding “physical burden” on the one hand, but patients with more expectations and therefore higher motivation to achieve improvements regarding “psychological burden” on the other hand, were more likely to have a better physical health status. As assumed before, less motivations and expectations regarding physical burden maybe due to a better general health outcome after disc surgery, which might have been reflected in this finding. Still, the reason why more rehabilitation-related motivations/expectations regarding “psychological burden” is associated with a better physical quality of life remains unclear.

On the other hand, a better mental quality of life was strongly associated with a better educational level (10th grade vs. 9th grade). This corresponds well with the study of Bjelland et al. [34] who found that low educational levels in adults are significantly associated with both depression and anxiety, whereas higher educational levels seem to have a protective effect. A better mental health status was weakly associated with less rehabilitation-related motivations/expectations regarding the need for “coping”, the relief of “physical burden” and with lower scores of “scepticism” towards rehabilitation treatment and moderately associated with less rehabilitation-related motivations/expectations regarding the relief of “psychological burden”. In addition, a better mental health status was weakly associated with more rehabilitation-related motivations/expectations regarding the improvement of “health”.

Patient satisfaction is an important outcome measure in rehabilitation research [35,36]. Thus, patients´ satisfaction seems to have an important impact on compliance regarding treatment, the keeping of appointments, the disclosure of important information, and commitment towards rehabilitation facility [37–39]. On the other hand, patients´ dissatisfaction may be linked to a reduction of treatment effectiveness, especially if rehabilitation activities are not fully attended or by a lack of compliance with prescribed treatment after discharge [39]. The present study found that the vast majority (>90%) of both IPR and OPR patients was satisfied with their rehabilitation treatment, which is well in line with the results of other studies [36,40,41]. A greater satisfaction with rehabilitation was weakly associated with male gender. This goes in line with Elliott et al. [42], who found that women reported generally less positive experiences than men regarding their hospital stay. Lillefjell [43] points out that knowledge about gender differences and the way psychosocial factors influence rehabilitation must be taken into account in designing rehabilitation intervention. The study could not find significant gender differences regarding pain intensity, pain experience, anxiety, depression and functional health status [43]. However, the findings suggest a moderating effect of gender regarding the sense of coherence response. Accordingly, in this study women reported significant lower manageability and comprehensibility scores compared to men [43]. Unlike to our results, previous works reported significant associations between older age and greater satisfaction with rehabilitation [36,39,44]. A reason for this might be that the age range of patients in the presented study was between 18 and 55 years. Age-related associations might therefore not become obvious as patients of older age groups were not part of the study sample. In addition, patients with higher motivations to achieve improvements regarding “psychological burden” were less likely to be satisfied with AHB. A possible explanation for this might be that patients who suffer from psychological burden have a greater interest to address their emotional problems during their rehabilitation treatment. As rehabilitation programs following herniated disc surgery might focus to a much greater degree to physical ailments, this might result in less satisfaction with AHB. Greater “knowledge” about rehabilitation treatment as an indicator for rehabilitation motivation also turned out to have positive influence on satisfaction with AHB. A practical implication from this finding might be, that increasing the level of rehabilitation-related information via information flyers or more detailed personal consultancy during acute hospital care may be favourable not only for patients´ treatment satisfaction but also for the overall rehabilitation outcome. Future studies should take a closer investigation of this matter into consideration.

Besides the longitudinal study design and a large sample size, a great strength of the study is that disc surgery patients were already approached during acute hospital treatment. Patients`individual answers regarding their motives, motivations and expectations regarding rehabilitation treatment and rehabilitation setting may therefore not be biased by social desirability towards rehabilitation clinic.

A limitation of the study is that the presented findings regarding the choice of a certain rehabilitation setting only rely on patient´s own reports regarding their preferences, motivations and expectations. This does not necessarily say anything about the concrete allocation procedure that lead to the decision for a certain rehabilitation setting. Hence, the physician´s opinion, the advice of the hospital social service and structural factors such as the lack of outpatient rehabilitation facilities in the patient´s neighbouring region may all contribute to the allocation procedure in different ways. Future studies should take all these allocation factors into account that allow making detailed conclusions on allocation procedures regarding different rehabilitation settings. Using mixed-method-designs of qualitative and quantitative survey techniques are strongly recommended in this context. Another limitation of the study is that, although taking patients´ pain intensity into account, it does not give information about the chronicity of neck and back pain problems and its influence on motivational and expectation factors. Regarding this matter, Skatteboe et al. [45] discuss that a prolonged duration of pain may reduce expectations for recovery. The authors argue that multiple examinations and treatments without concrete improvements may lead to a decline in motivation and participation. In this context longitudinal studies are needed to investigate this complex matter.

## Conclusions

Little is known about the role of patients´ motivations and expectations regarding the choice of an inpatient or outpatient rehabilitation setting. The present study, for the first time, provides information about individual motives, motivations and expectations behind this decision in a sample of patients undergoing herniated disc surgery. Thus, this study contributes to a better understanding of the patients`perspective within decision-making process for a specific rehabilitation setting. Rehabilitation-related motivations and expectations before rehabilitation had impact on physical and mental quality of life as well as on satisfaction with rehabilitation treatment three months after acute hospital stay. There were also several other associations with rehabilitation outcome parameters: Thus, higher age, a better educational level, the absence of other chronic diseases, a shorter length of acute care hospital stay, a better subjective prognosis of gainful employment and OPR were associated with improved ability to work. Male gender, the absence of other chronic diseases, lower pain intensity, a shorter length of acute care hospital stay, employment within the last three months, a better subjective prognosis of gainful employment and OPR was significantly associated with a better physical health status. A higher educational level, a lumbar disc herniation and the absence of psychiatric comorbidity before surgery were significantly associated with a better mental health status. In addition, male gender was associated with a greater satisfaction with rehabilitation. The present study therefore suggests the following clinical implications for patients undergoing herniated disc surgery. First, the assessment of rehabilitation-related motives, motivations and expectations is strongly recommended at the beginning of rehabilitation treatment, preferably already at the end of the preceding acute hospital stay. Taking motivational and expectation-related aspects of patients undergoing herniated disc surgery into account at an early stage may help to find the perfect match between rehabilitation patient and rehabilitation setting. Another advantage of such an assessment could be that special motives, such as the need for psychological counselling, could be addressed during rehabilitative care and would be prevented from being overseen. Second, increasing the level of information regarding similarities and differences of IPR and OPR on the one hand and providing material regarding general treatment procedures on the other hand might help to improve allocation processes and satisfaction with rehabilitation. Third, assessing patients´ self-prediction of return to work after disc surgery on a regular basis and in the same context individual counselling regarding realistic goal setting may help to improve work-related rehabilitation outcome. Fourth, the establishment of self-help groups may increase feelings of connectedness and social support, especially for female patients, and may help to reduce gender differences regarding health-related quality of life. Longitudinal studies incorporating qualitative and quantitative methods are strongly recommended to investigate if these measures help to improve rehabilitation outcomes.
